# Drug resistance and genetic transmission characteristics of HIV-1 CRF55_01B in people living with HIV/AIDS (PLWHA) in Henan Province, China

**DOI:** 10.1186/s12977-025-00665-2

**Published:** 2025-05-29

**Authors:** Jie Ma, Jinjin Liu, Shuguang Wei, Mingjie Hou, Qingxia Zhao, Yuqi Huo

**Affiliations:** Affiliated Infectious Diseases Hospital of Zhengzhou University (Henan Infectious Diseases Hospital, The Sixth People’s Hospital of Zhengzhou), Center for Translational Medicine, Zhengzhou, 450000 People’s Republic of China

**Keywords:** Drug resistance mutations, Pretreatment drug resistance, Acquired drug resistance, Transmission cluster

## Abstract

**Background:**

Among the many CRFs, CRF55_01B was the first CRF01_AE and subtype B recombinant strain identified around 2013 among men who have sex with men (MSM) in Shenzhen, China. With rapid spreading throughout the country, CRF55_01B has attracted much attention in recent years. This study aimed to analyze its prevalence of drug resistance and transmission characteristics in people living with HIV/AIDS (PLWHA) in Henan province, China so as to pay particular attention to this group of individuals to reduce the incidence of drug resistance.

**Results:**

Two hundred and forty-five CRF55_01B-infected individuals, including 141 treatment-naïve and 104 treatment-experienced individuals, were enrolled. In treatment-naïve individuals, 6.38% (9/141) of them harboured NRTI DRMs and 19.15% (27/141) of them harboured NNRTI DRMs except V179E/D. In treatment-experienced individuals, 2.00% (2/100) harboured INSTI DRMs, 82.69% (86/104) of them harboured NRTI DRMs, and 88.46% (92/104) of them harboured NNRTI DRMs except V179E/D. The overall prevalence of ADR was 89.42% (93/104), while the prevalence of PDR was 19.86% (28/141). A total of 23 transmission clusters, accounting for 37.55% (92/245) of the total sequences, were identified. The clusters ranged in size from 2 to 19, and 15 (65.22%) had 3 or more sequences.

**Conclusions:**

High prevalence of DRMs and drug resistance were observed in CRF55_01B in both treatment-naïve and treatment-experienced individuals, particular attention should be paid to this group of individuals to reduce the incidence of drug resistance.

**Supplementary Information:**

The online version contains supplementary material available at 10.1186/s12977-025-00665-2.

## Background

Human immunodeficiency virus type 1 (HIV-1), belonging to the lentivirus genus of the *Retroviridae* family, is a positive strand RNA virus. Due to high replication capacity and poor fidelity of its reverse transcriptase, HIV-1 evolves rapidly. Since the emergence of the prototype HIV-1 virus, which was designated as subtype B, many novel subtypes and recombinants have been identified. Globally, the proportion of recombinants increases rapidly. In China, based on national molecular epidemiological survey, 10 subtypes and more than 20 circulating recombinant forms (CRFs) among people living with HIV/AIDS (PLWHA) have been identified [1–3]. Currently, CRFs, namely CRF01_AE and CRF07_BC, have replaced subtype B as the dominant variants in newly diagnosed cases in China [4]. Among the many CRFs, CRF55_01B was the first CRF01_AE and subtype B recombinant strain identified around 2013 among men who have sex with men (MSM) in Shenzhen, China [5]. The estimated time of the most recent common ancestor (tMRCA) indicated that CRF55_01B emerged around 2000–2003 and mainly spread in MSM population [6]. With rapid spreading throughout the country and spilling from MSM population to heterosexual population, CRF55_01B has attracted much attention in recent years [6–8].

Based on molecular network and Bayesian correlation analysis, CRF55_01B was proposed to begin to spread to other provinces such as Henan after 2010 [6]. The prevalence of CRF55_01B among treatment-naïve individuals was 6.12% in Henan Province during Jan, 2022 to Feb, 2023, making it the fourth prevalent strain right after CRF07_BC, CRF01_AE and subtype B [9]. The study of Wei et al. showed that CRF55_01B-infected individuals had higher plasma viral load (VL) than CRF01_AE and CRF07_BC at the initiation of antiretroviral treatment (ART) [10]. Henan Province is one of the top six high-prevalence provinces for HIV [4]. It has more than 71,000 PLWHA and 3596 newly diagnosed cases were reported in 2022 [9]. The increasing prevalence of CRF55_01B in Henan Province warrants the necessity of further study of its transmission and drug resistance profiles. Therefore, in this study we conducted a detailed analysis of DRMs and molecular network in CRF55_01B-infected individuals in Henan Province.

## Material and methods

### Participants enrollment

PLWHA who visited the Sixth People’s Hospital of Zhengzhou from January 2018 to December 2023 for routine surveillance of drug resistance were subjected to an In-house genotypic drug resistance testing. The HIV-1 partial pol gene fragments (HXB2 2253-3353) were reverse-transcribed and amplified as described previously [11]. The subtype of HIV isolates was analyzed using REGA HIV-1 Subtyping Tool (http://dbpartners.stanford.edu:8080/RegaSubtyping/stanford-hiv/typingtool/) based on the partial *pol* region and further confirmed by phylogenetic analysis. A phylogenetic tree was constructed using Molecular Evolutionary Genetic Analysis (MEGA) software (version X) based on the most suitable model (General Time Reversible plus Gamma model) determined using the FindModel tool (https://www.hiv.lanl.gov/content/sequence/findmodel/findmodel.html). The full-length integrase (INT) gene fragment (HXB2 4230-5093) was also reverse-transcribed and amplified. The procedure for the amplification of the target fragments was performed as described previously [12]. Demographic data and medical records, including age, sex, transmission route, and treatment regimen, were collected.

### Drug resistance mutation and drug resistance analysis

Stanford HIV drug resistance database (http://hivdb.stanford.edu/) was used for DRMs analysis and DRMs in accordance with the World Health Organization surveillance list for nucleoside reverse transcriptase inhibitors (NRTIs), non-nucleoside reverse transcriptase inhibitors (NNRTIs), and protease inhibitors (PIs) were included in subsequent analysis. As there is no reference list for integrase strand transfer inhibitors (INSTIs), INSTI-associated mutations provided by the Stanford HIV DRM database were adopted in this study. The algorithm for the estimation of drug resistance is that each DRM is given a penalty score and the estimated level of resistance to a drug is determined by adding up the penalty scores associated with each of the DRMs present in a sequence. Once the total score is calculated the estimated level of resistance can be calculated as follows: susceptible (total score 0 to 9); potential low-level resistance (total score 10 to 14); low-level resistance (total score 15 to 29); intermediate resistance (total score 30 to 59); and high-level resistance (total score ≥ 60). For individual DRMs, only those that give a score at or greater than 15 were calculated.

### Transmission network analysis

All partial pol gene sequences were aligned using MEGA software (version XI). The aligned sequences were used to calculate genetic distances between sequence pairs based on the Tamura-Nei 93 model incorporated in HyPhy 2.2.4. A genetic distance of ≤ 1.5% between the sequence pairs indicated that they were transmission partners [13, 14]. The transmission network was constructed using Cytoscape software (version 3.5.1) for visualization.

### Sequence data

The gene sequences were submitted to GenBank (Supplementary Table 1). The sampling years were also displayed in Supplementary Table 1.

## Results

### Basic information

Based on subtyping and phylogenetic analysis (Supplementary Fig. 1), a total of 245 individuals (141 treatment-naïve and 104 treatment-experienced) were classified as infected with CRF55_01B during the study period. Of these individuals, 236 (136 treatment-naïve and 100 treatment-experienced) full-length INT gene sequences were also successfully amplified. The age of them ranged from 17 to 76 years, with a median age of 36 years. 94.29% of them were men (231/245), and 44.08% of them were married. Among the main routes of infection, men who have sex with men (MSM) was the predominant transmission route (46.53%, 114/245), followed by heterosexual (HET) (31.84%, 78/245). Detailed characteristics of these CRF55_01B-infected individuals are shown in Table 1.

**Table 1:** Demographic characteristics of individuals infected with CRF55_01B

	All (n = 245)	Treatment-naïve (n = 141)	Treatment-experienced (n = 104)
Sex, n (%)			
Male	231 (94.29)	130 (92.20)	101 (97.12)
Female	14 (5.71)	11 (7.80)	3 (2.88)
Age (years),median (range)	36 (17–76)	35 (17–74)	37 (21–76)
< 18 years, n (%)	3 (1.21)	3 (2.13)	0 (0.00)
18–50 years, n (%)	196 (80.00)	109 (77.30)	87 (83.65)
> 50 years, n (%)	46 (18.78)	29 (20.57)	17 (16.35)
Marital status, n (%)			
Single	74 (30.02)	57 (40.43)	17 (16.35)
Married	108 (44.08)	66 (46.81)	42 (40.38)
Divorced/widowed	20 (8.16)	9 (6.38)	11 (10.58)
Unknown	43 (17.55)	9 (6.38)	34 (32.69)
Route groups, n (%)			
MSM	114 (46.53)	74 (52.48)	40 (38.46)
HST	78 (31.84)	47 (33.33)	31 (29.81)
OTH	53 (21.63)	20 (14.18)	33 (31.13)
CD4+ T cell count (cells/μl)			
CD4+, median (minimum–maximum)	79 (1/786)	106 (1/531)	34 (1/786)
Initial treatment regimen, n (%)			
TDF + 3TC + EFV	72 (29.39)	–	72 (69.23)
AZT + 3TC + EFV	7 (2.86)	–	7 (6.73)
TDF + 3TC + NVP	5 (2.04)	–	5 (4.81)
AZT + 3TC + NVP	2 (0.82)	–	2 (1.92)
TDF + 3TC + LPV/r	0 (0.00)	–	0 (0.00)
Other	1 (0.41)	–	1 (0.96)
Unkown	17 (6.94)	–	17 (16.35)
Treatment naïve	141 (57.55)	141 (100)	–

*MSM* men who have sex with men, *HST* heterosexual orientation, *OTH* others, including patients whose risk factors were unknown or patients who did not provide information

### Prevalence and distribution of DRMs

**Fig. 1:**
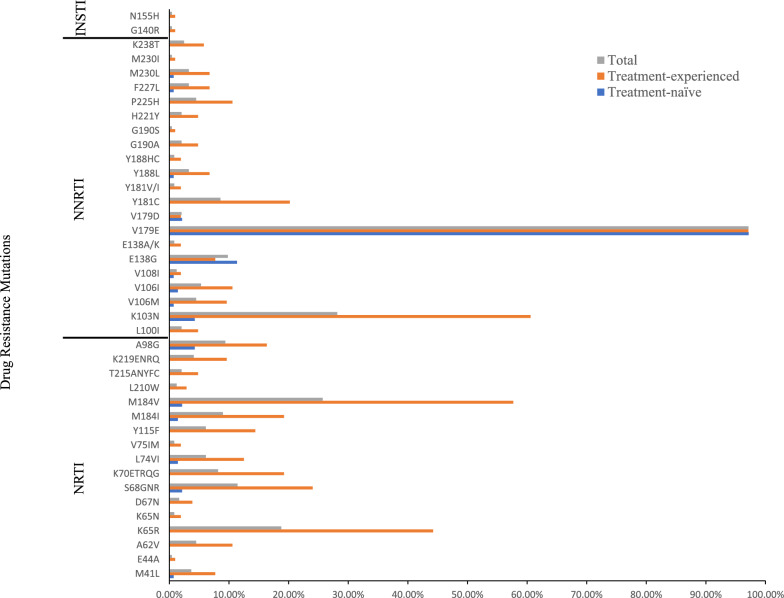
The percentage of DRMs in the CRF55_01B-infected individuals in the treatment-naïve and treatment-experienced groups

Of the 245 CRF55_01B-infected individuals, 50.20% (123/245), namely, 21.28% (30/141) of the treatment-naïve individuals and 89.42% (93/104) of the treatment-experienced individuals, carried at least one DRM except V179E/D, which was present in all but two of them. In treatment-naïve individuals, 6.38% (9/141) of them harboured NRTI DRMs, 19.15% (27/141) of them harboured NNRTI DRMs, and none of them harboured PI and INSTI DRMs. Among the NRTI DRMs, M184V/I (3.55%, 5/141) was the most frequent, followed by S68G (2.13%, 3/141). Among the NNRTI DRMs, the most frequent mutation was E138G (11.35%, 16/141), followed by A98G (4.26%, 6/141) and K103N (4.26%, 6/141) (Fig. 1).

In treatment-experienced individuals, 82.69% (86/104) of them harboured NRTI DRMs, 88.46% (92/104) of them harboured NNRTI DRMs, 2.00% of them (2/100) harboured INSTI DRMs, and none of them harboured PI DRMs. Among the NRTI DRMs, M184V/I (76.92%, 80/104) was the most frequent, followed by K65R (44.23%, 46/104), S68G/N/R (24.04%, 25/104), and K70 (19.23%, 20/104). Among the NNRTI DRMs, the most frequent mutation was K103N (60.58%, 63/104), followed by V106M/I (20.19%, 21/104), and Y181C (20.19%, 21/104) (Fig. 1). Dual-class mutations, namely NRTI plus NNRTI resistance mutations, were detected in 82.69% (86/104) of these individuals. The most common combination of mutations was M184V/I + K103N, with a frequency of 52.88% (55/104), followed by M184V/I + K65R, with a frequency of 39.42% (41/104). Triple-class mutations were found in one patient.

### Drug resistance

**Fig. 2:**
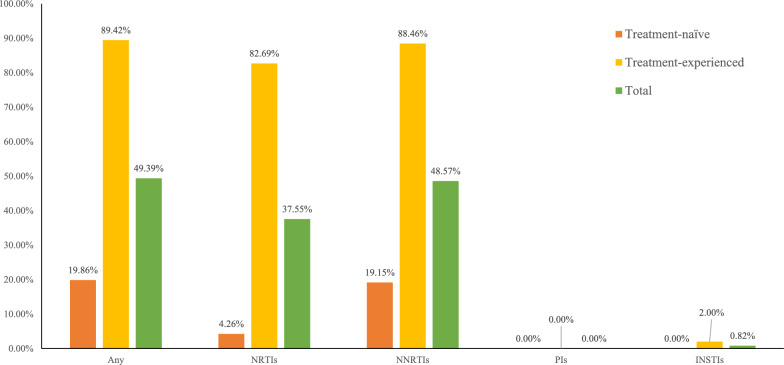
The percentage of drug resistance in the CRF55_01B-infected individuals in the treatment-naïve and treatment-experienced groups

Among the 245 individuals, DRMs associated with low-level or higher levels resistance to any drug was detected in 49.39% (121/245): NRTIs (37.55%, 92/245), NNRTIs (48.57%, 119/245) and INSTIs (0.85%, 2/236). The prevalence of pretreatment drug resistance (PDR) was 19.86% (28/141), while the overall prevalence of acquired drug resistance (ADR) was 89.42% (93/104). In treatment-naïve individuals, drug resistance to NRTIs and NNRTIs accounted for 4.26% (6/141) and 19.15% (27/141), respectively, and drug resistance to PIs and INSTIs were not detected. In treatment-experienced individuals, drug resistance to NRTIs, NNRTIs, and INSTIs accounted for 82.69% (86/104), 88.46% (92/104), and 2.00% (2/100), respectively, and resistance to PIs was not detected (Fig. 2).

**Fig. 3:**
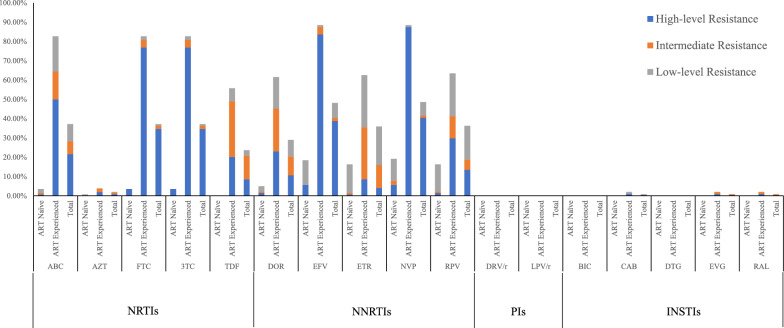
Different drug resistance levels of four classes of antiretroviral drugs predicted by the Stanford HIVdb Program the CRF55_01B-infected individuals in the treatment-naïve and treatment-experienced groups. *ABC* abacavir, *AZT* zidovudine, *FTC* emtricitabine, *3TC* lamivudine, *TDF* tenofovir, *DOR* doravirine, *EFV* efavirenz, *ETR* etravirine, *NVP* nevirapine, *RPV* rilpivirine, *DRV/r* darunavir/r, *LPV/r* lopinavir/r, *BIC* bictegravir, *CAB* cabotegravir, *DTG* dolutegravir, *EVG* elvitegravir, *RAL* raltegravir

Resistance to commonly used drugs in clinical settings was further analyzed. Intermediate- to high-level resistance to lamivudine (3TC) and emtricitabine (FTC) of the NRTIs was the most prevalent in treatment-experienced individuals, followed by abacavir (ABC), and the same pattern was observed in treatment-naïve individuals. For NNRTIs, intermediate- to high-level resistance to nevirapine (NVP) was the most commonly observed in both treatment-experienced and treatment-naive individuals, followed by efavirenz (EFV) and rilpivirine (RPV). For INSTIs, resistance to cabotegravir (CAB), raltegravir (RAL), and elvitegravir (EVG) was observed in treatment-experienced individuals (Fig. 3).

### Transmission characteristics

**Fig. 4:**
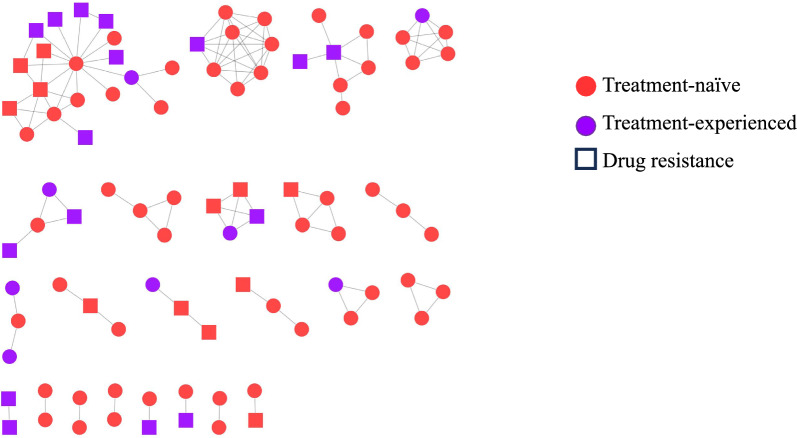
The transmission networks of CRF55_01B-infected individuals. Different colors are used to represent treatment-naïve and treatment-experienced, respectively. Rectangle represents drug resistance

Considering the high-level of PDR and ADR in CRF55_01B-infected individuals, we further performed molecular network analysis to determine if individuals with PDR could be traced to individuals with ADR. Under the threshold of 1.5% genetic distance, sequences from both treatment-naïve and treatment-experienced individuals formed a total of 23 transmission clusters (TCs), accounting for 37.55% (92/245) of the total sequences. These TCs ranged in size from 2 to 19, and 15 (65.22%) had 3 or more sequences. Drug resistance was identified in 13 TCs and 7 of these TCs (47 sequences) contained both treatment-naïve and treatment-experienced individuals (Fig. 4).

## Discussion

The CRF55_01B originated from MSM has increased rapidly in recent years and was detected in all provinces of China [15]. It showed varied prevalence in different regions, such as 4.1% in Tianjin [16], 4.7% in Xi’an [17], 6.3% in Hefei [15], 10.30% in Guangdong Province [18], and 12.96% in Guangxi Province [19]. In Henan Province, CRF55_01B was the fourth prevalent subtype right after CRF07_BC, CRF01_AE and subtype B among treatment-naïve individuals [9]. Its rapid transmission may be attributed to the rapid development of transportation and technology [6]. In this study we first analyzed the characteristics of PDR and ADR among CRF55_01B-infected individuals. Our results showed that 28 individuals with drug resistance were identified among 141 treatment-naïve individuals, giving a PDR rate of 19.86%, significantly higher than that (9.24%, 11/119) in Guangdong Province [20] and also higher than those of other genotypes, such as CRF07_BC (5.26%, 14/266) and CRF01_AE (13.89%, 25/180) in Henan Province [9]. PDR is critical to the first-line ART options and the reduction of drug resistance, as which may lead to higher transmission risk. WHO’s new recommendations suggest that countries displaying a population-prevalence of PDR above 10% should urgently consider non-NNRTI first-line ART regimens, such as integrase inhibitors [21]. Drug resistance was identified in 93 of 104 treatment-experienced individuals, giving an ADR rate of 89.42%, which was higher than that (79.01%, 128/162) in Guangdong Province [22] and much higher than the overall ADR rate (44.7%) in China [23].

Further analysis showed varied resistance mutation profiles of CRF55_01B in treatment-experienced and treatment-naïve individuals. For NRTI mutations, higher percentages of M184V/I and K65R were detected in treatment-experienced individuals when compared with treatment-naïve individuals, with 76.92% versus 3.55% and 44.32% versus 0%, respectively. M184V/I cause high-level resistance to lamivudine and emtricitabine, and low-level resistance to abacavir [24, 25]. K65R causes intermediate-level resistance to abacavir and tenofovir [24]. However, M184 plus K65R increases resistance to abacavir. The impact of M184 plus K65R for CRF55_01B-infected individuals need to be continuously evaluated because of their high frequency (39.42%, 41/104) observed in this study, which was much higher than that (14.81%, 24/162) observed in Guangdong [22]. For NNRTI mutations, V179 was present in all but two of the 245 individuals infected with CRF55_01B, which is consistent with the results of previous studies [7, 22]. V179E is a natural mutation and contributes to potential low-level drug resistance to EFV and NVP, but when it is combined with other drug-resistant mutations such as E138G, it reduces the effectiveness of most NNRTIs [7, 26]. In treatment-experienced individuals, K103N was another dominant prevalent resistance mutation (60.58%). Particularly, K103N was generally accompanied with Y181C, which might be related to the facts that more than 70% of individuals were exposed to EFV-based regimens. K103N plus M184V/I mutations can lead to failure of regimens comprising FTC, 3TC, EFV, or NVP [27] and more than 50% individuals with DRMs carried both mutations in this study. In treatment-naïve individuals, E138G, secondary to V179E, was another dominant prevalent resistance mutation (11.35%) which causes low-level drug resistance to RPV and potential low-level drug resistance to ETR, EFV, and NVP [24]. PI-related major resistance mutation was not detected in all individuals, and INSTI resistance mutation was detected only in two treatment-experienced individuals. To further confirm if these individuals are genetically linked, transmission networks based on partial *pol* gene sequences were constructed. A total of 23 TCs, containing over 1/3 of the total sequences, were identified and in the TCs (56.52%, 13/23) involving both treatment-naïve and treatment-experienced individuals, drug resistance were primarily identified in treatment-experienced individuals, suggesting possible acquirement of those DRMs during treatment.

The primary limitation of this study is that based on the prevalence of CRF55_01B (~ 6%) and annual incidence of HIV (approximately 4500 cases) in Henan Province, there are approximately 270 newly CRF55_01B-infected individuals each year and only limited number of cases were included during the study period, thus the actual PDR and ADR could not be accurately reflected. Secondly, the limited numbers of cases also compromised our transmission network analysis.

## Conclusions

In this study high prevalence of DRMs and drug resistance were observed in CRF55_01B in both treatment-naïve and treatment-experienced individuals in Henan Province, particular attention should be paid to this group of individuals to reduce the incidence of drug resistance.

## Supplementary Information

Supplementary Material 1.

Supplementary Material 2.

## Data Availability

No datasets were generated or analysed during the current study.

## Abbreviations

CRFs: Circulating recombinant forms
DRMs: Drug resistance mutations
PDR: Pre-treatment drug resistance
ADR: Acquired drug resistance
NRTIs: Nucleoside reverse transcriptase inhibitors
NNRTIs: Non-nucleoside reverse transcriptase inhibitors
PIs: Protease inhibitors
INSTIs: Integrase strand transfer inhibitors
TCs: Transmission clusters

## References

[1] Fan Q, Zhang J, Luo M, et al. Molecular genetics and epidemiological characteristics of HIV-1 epidemic strains in various sexual risk behaviour groups in developed Eastern China, 2017–2020. Emerg Microbes Infect. 2022;11(1):2326–39. 36032035 10.1080/22221751.2022.2119167 PMC9542350

[2] Yanling L, Yi F, Shao Y. The origin and molecular epidemiology of HIV-1 subtype C, CRF07_BC and CRF08_BC. Chin J AIDS STD. 2021;27(05):549–52.

[3] Acquired Immunodeficiency Syndrome and Hepatitis C Professional Group Society of Infectious Diseases, Chinese Medical Association, Chinese Center for Disease Control and Prevention. Chinese guidelines for diagnosis and treatment of human immunodeficiency virus/acquired immunodeficiency syndrome. Chin J Infect Dis. 2021;39(12):715–35.

[4] Vrancken B, Zhao B, Li X, et al. Comparative circulation dynamics of the five main HIV types in China. J Virol. 2020;94(23):e00683-e1620. 32938762 10.1128/JVI.00683-20 PMC7654276

[5] Zhao J, Cai W, Zheng C, et al. Origin and outbreak of HIV-1 CRF55_01B among MSM in Shenzhen, China. J Acquir Immune Defic Syndr. 2014;66(3):e65-67. 24662297 10.1097/QAI.0000000000000144

[6] Gan M, Zheng S, Hao J, et al. The prevalence of CRF55_01B among HIV-1 strain and its connection with traffic development in China. Emerg Microbes Infect. 2021;10(1):256–65. 33512306 10.1080/22221751.2021.1884004 PMC7894451

[7] Liu Y, Li H, Wang X, et al. Natural presence of V179E and rising prevalence of E138G in HIV-1 reverse transcriptase in CRF55_01B viruses. Infect Genet Evol. 2020;77: 104098. 31678241 10.1016/j.meegid.2019.104098

[8] Zhou PP, Yu G, Kuang YQ, et al. Rapid and complicated HIV genotype expansion among high-risk groups in Guangdong Province, China. BMC Infect Dis. 2019;19(1):185. 30795762 10.1186/s12879-019-3788-7 PMC6387515

[9] Liu J, Liu C, Wang Y, et al. Increased prevalence of pretreatment drug resistance mutations in treatment-naïve people living with HIV-1 in Henan Province, China (2022/23). Infect Genet Evol. 2023;115: 105520. 37898414 10.1016/j.meegid.2023.105520

[10] Wei L, Li H, Lv X, et al. Impact of HIV-1 CRF55_01B infection on the evolution of CD4 count and plasma HIV RNA load in men who have sex with men prior to antiretroviral therapy. Retrovirology. 2021;18(1):22. 34399785 10.1186/s12977-021-00567-z PMC8365277

[11] Yang Z, Wei S, Liu J, et al. Characterization of HIV-1 subtypes and drug resistance mutations in Henan Province, China (2017–2019). Arch Virol. 2020;165(6):1453–61. 32279138 10.1007/s00705-020-04606-6 PMC7222071

[12] Yang Z, Yang X, Deng X, et al. Prevalence of integrase strand transfer inhibitor (INSTIs) resistance mutations in Henan Province, China (2018–2020). Infection. 2021;49(6):1195–202. 34279816 10.1007/s15010-021-01668-9

[13] Hassan AS, Pybus OG, Sanders EJ, Albert J, Esbjörnsson J. Defining HIV-1 transmission clusters based on sequence data. AIDS. 2017;31(9):1211–22. 28353537 10.1097/QAD.0000000000001470 PMC5482559

[14] Oster AM, Wertheim JO, Hernandez AL, Ocfemia MC, Saduvala N, Hall HI. Using molecular HIV surveillance data to understand transmission between subpopulations in the United States. J Acquir Immune Defic Syndr. 2015;70(4):444–51. 26302431 10.1097/QAI.0000000000000809 PMC4878401

[15] Zheng S, Wu J, Hao J, et al. Epidemic characteristics of HIV drug resistance in Hefei, Anhui Province. Pathogens. 2022;11(8):866. 36014987 10.3390/pathogens11080866 PMC9416635

[16] Zheng M, Yu M, Cheng S, et al. Characteristics of HIV-1 molecular transmission networks and drug resistance among men who have sex with men in Tianjin, China (2014–2018). Virology journal. 2020;17(1):169. 33143744 10.1186/s12985-020-01441-8 PMC7640427

[17] Xia H, Jin J, Ba H, et al. Genetic diversity and characteristics of drug resistance among treatment-naive people living with HIV in Xi’an, China. Drug Des Dev Ther. 2023;17:1485–94. 10.2147/DDDT.S406255 PMC10200113 37220545

[18] Lan Y, Li L, He X, et al. Transmitted drug resistance and transmission clusters among HIV-1 treatment-naïve patients in Guangdong, China: a cross-sectional study. Virol J. 2021;18(1):181. 34488793 10.1186/s12985-021-01653-6 PMC8422730

[19] Pang X, Xie B, He Q, et al. Distinct rates and transmission patterns of major HIV-1 subtypes among men who have sex with men in Guangxi. China Front Microbiol. 2023;14:1339240. 38282731 10.3389/fmicb.2023.1339240 PMC10822680

[20] Yu G, Li Y, Huang X, et al. Genetic diversity and drug resistance of HIV-1 CRF55_01B in Guangdong. China Curr HIV Res. 2020;18(3):210–8. 32294040 10.2174/1570162X18666200415140652

[21] WHO. Guidelines on the public health response to pretreatment HIV drug resistance. Geneva: World Health Organization; 2017.

[22] Lan Y, Xin R, Cai W, et al. Characteristics of drug resistance in HIV-1 CRF55_01B from ART-experienced patients in Guangdong, China. J Antimicrob Chemother. 2020;75(7):1925–31. 32300784 10.1093/jac/dkaa116

[23] Zuo L, Liu K, Liu H, et al. Trend of HIV-1 drug resistance in China: a systematic review and meta-analysis of data accumulated over 17 years (2001–2017). EClinicalMedicine. 2020;18: 100238. 31922125 10.1016/j.eclinm.2019.100238 PMC6948268

[24] Zhang M, Ma Y, Wang G, et al. The profile of HIV-1 drug resistance in Shanghai, China: a retrospective study from 2017 to 2021. J Antimicrob Chemother. 2024;79(3):526–30. 38300833 10.1093/jac/dkad370 PMC10904715

[25] Frost SD, Nijhuis M, Schuurman R, et al. Evolution of lamivudine resistance in human immunodeficiency virus type 1-infected individuals: the relative roles of drift and selection. J Virol. 2000;74(14):6262–8. 10864635 10.1128/jvi.74.14.6262-6268.2000 PMC112131

[26] Pang X, Liang S, Tang K, et al. Disparity of HIV-1 pretreatment drug resistance in men who have sex with men and the heterosexual population in Guangxi, China. Open Forum Infect Dis. 2023;10(2):ofad016. 36751650 10.1093/ofid/ofad016 PMC9898876

[27] Melikian GL, Rhee SY, Varghese V, et al. Non-nucleoside reverse transcriptase inhibitor (NNRTI) cross-resistance: implications for preclinical evaluation of novel NNRTIs and clinical genotypic resistance testing. J Antimicrob Chemother. 2014;69(1):12–20. 23934770 10.1093/jac/dkt316 PMC3861329

