# Supplementary material for: Drug resistance and genetic transmission characteristics of HIV-1 CRF55_01B in people living with HIV/AIDS (PLWHA) in Henan Province, China
Source: Retrovirology. 2025 May 29;22:9. doi: 10.1186/s12977-025-00665-2 (PMC12121082; doi:10.1186/s12977-025-00665-2)
Supplement: Supplementary file 1 — Supplementary Material 1. [file 12977_2025_665_MOESM1_ESM.pptx]

## Slide 1
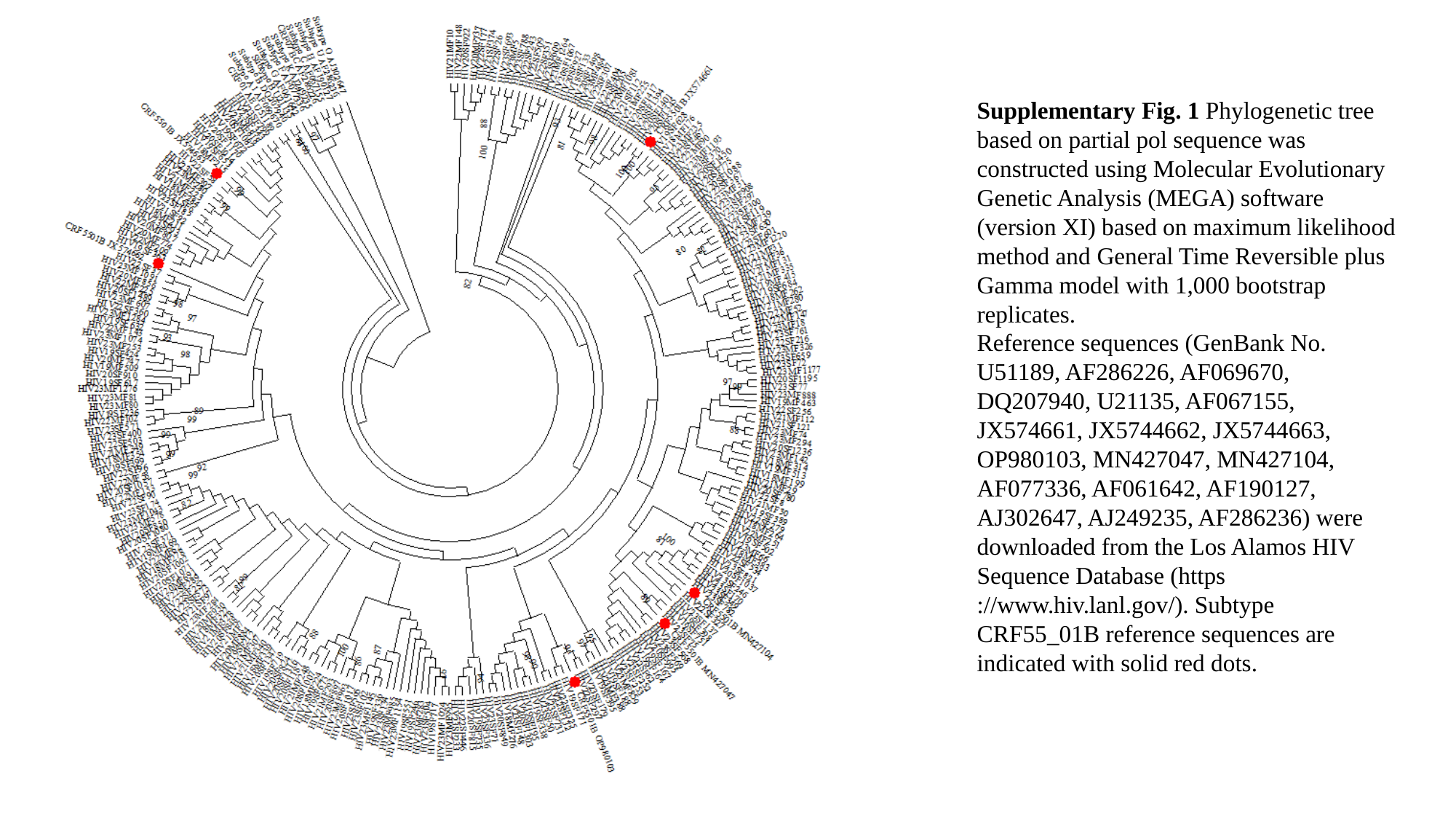

Supplementary Fig. 1 Phylogenetic tree based on partial pol sequence was
constructed using Molecular Evolutionary Genetic Analysis (MEGA) software (version XI) based on maximum likelihood
method and General Time Reversible plus Gamma model with 1,000 bootstrap replicates.
Reference sequences (GenBank No. U51189, AF286226, AF069670, DQ207940, U21135, AF067155, JX574661, JX5744662, JX5744663, OP980103, MN427047, MN427104, AF077336, AF061642, AF190127, AJ302647, AJ249235, AF286236) were downloaded from the Los Alamos HIV Sequence Database (https ://www.hiv.lanl.gov/). Subtype CRF55_01B reference sequences are indicated with solid red dots.
