# Supplementary material for: Drug resistance and genetic transmission characteristics of HIV-1 CRF55_01B in people living with HIV/AIDS (PLWHA) in Henan Province, China
Source: Retrovirology. 2025 May 29;22:9. doi: 10.1186/s12977-025-00665-2 (PMC12121082; doi:10.1186/s12977-025-00665-2)
Supplement: Supplementary file 2 — Supplementary Material 2. [file 12977_2025_665_MOESM2_ESM.pptx]

## Slide 1
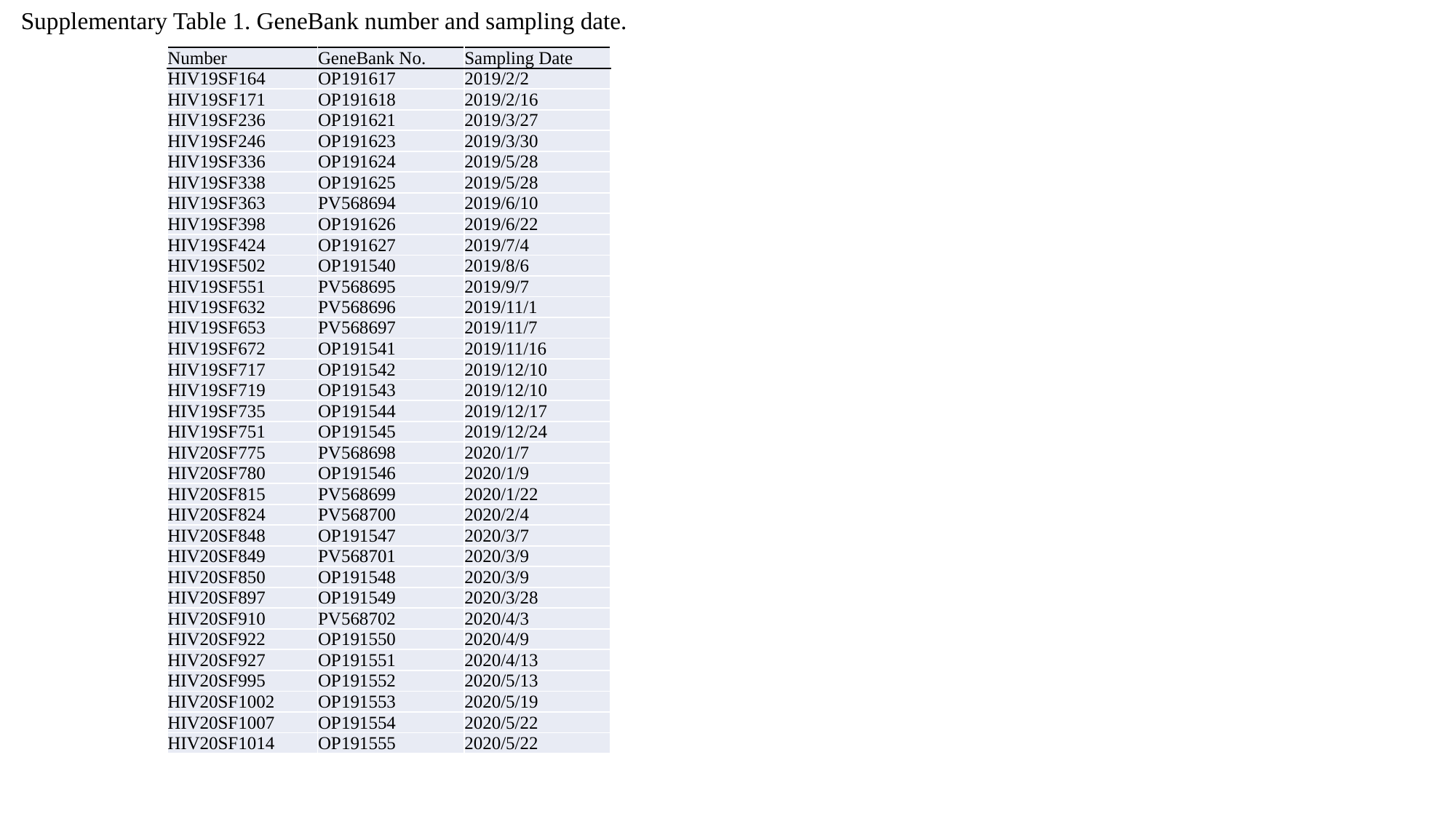

Supplementary Table 1. GeneBank number and sampling date.
| Number | GeneBank No. | Sampling Date |
| --- | --- | --- |
| HIV19SF164 | OP191617 | 2019/2/2 |
| HIV19SF171 | OP191618 | 2019/2/16 |
| HIV19SF236 | OP191621 | 2019/3/27 |
| HIV19SF246 | OP191623 | 2019/3/30 |
| HIV19SF336 | OP191624 | 2019/5/28 |
| HIV19SF338 | OP191625 | 2019/5/28 |
| HIV19SF363 | PV568694 | 2019/6/10 |
| HIV19SF398 | OP191626 | 2019/6/22 |
| HIV19SF424 | OP191627 | 2019/7/4 |
| HIV19SF502 | OP191540 | 2019/8/6 |
| HIV19SF551 | PV568695 | 2019/9/7 |
| HIV19SF632 | PV568696 | 2019/11/1 |
| HIV19SF653 | PV568697 | 2019/11/7 |
| HIV19SF672 | OP191541 | 2019/11/16 |
| HIV19SF717 | OP191542 | 2019/12/10 |
| HIV19SF719 | OP191543 | 2019/12/10 |
| HIV19SF735 | OP191544 | 2019/12/17 |
| HIV19SF751 | OP191545 | 2019/12/24 |
| HIV20SF775 | PV568698 | 2020/1/7 |
| HIV20SF780 | OP191546 | 2020/1/9 |
| HIV20SF815 | PV568699 | 2020/1/22 |
| HIV20SF824 | PV568700 | 2020/2/4 |
| HIV20SF848 | OP191547 | 2020/3/7 |
| HIV20SF849 | PV568701 | 2020/3/9 |
| HIV20SF850 | OP191548 | 2020/3/9 |
| HIV20SF897 | OP191549 | 2020/3/28 |
| HIV20SF910 | PV568702 | 2020/4/3 |
| HIV20SF922 | OP191550 | 2020/4/9 |
| HIV20SF927 | OP191551 | 2020/4/13 |
| HIV20SF995 | OP191552 | 2020/5/13 |
| HIV20SF1002 | OP191553 | 2020/5/19 |
| HIV20SF1007 | OP191554 | 2020/5/22 |
| HIV20SF1014 | OP191555 | 2020/5/22 |

## Slide 2
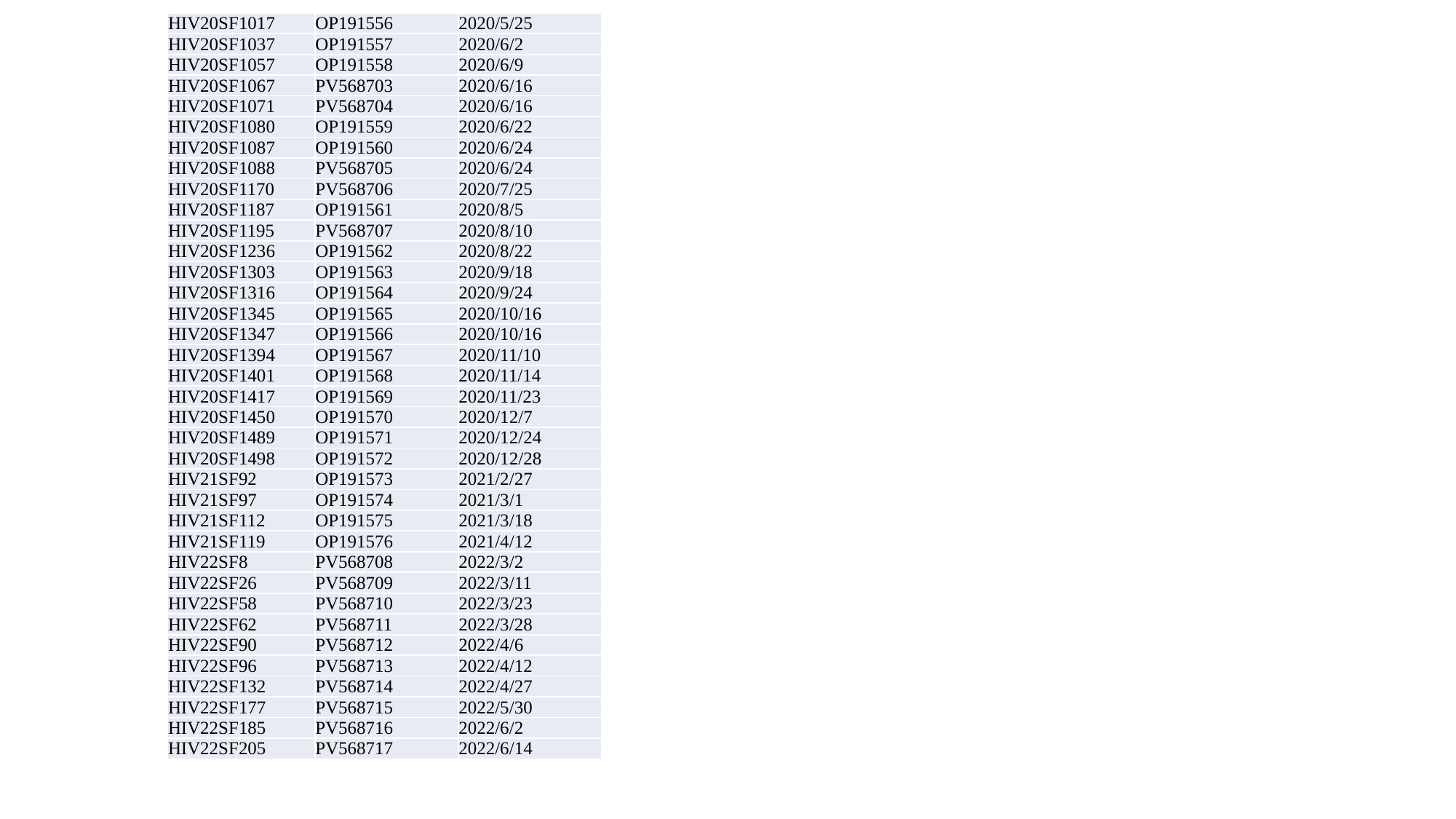

| HIV20SF1017 | OP191556 | 2020/5/25 |
| --- | --- | --- |
| HIV20SF1037 | OP191557 | 2020/6/2 |
| HIV20SF1057 | OP191558 | 2020/6/9 |
| HIV20SF1067 | PV568703 | 2020/6/16 |
| HIV20SF1071 | PV568704 | 2020/6/16 |
| HIV20SF1080 | OP191559 | 2020/6/22 |
| HIV20SF1087 | OP191560 | 2020/6/24 |
| HIV20SF1088 | PV568705 | 2020/6/24 |
| HIV20SF1170 | PV568706 | 2020/7/25 |
| HIV20SF1187 | OP191561 | 2020/8/5 |
| HIV20SF1195 | PV568707 | 2020/8/10 |
| HIV20SF1236 | OP191562 | 2020/8/22 |
| HIV20SF1303 | OP191563 | 2020/9/18 |
| HIV20SF1316 | OP191564 | 2020/9/24 |
| HIV20SF1345 | OP191565 | 2020/10/16 |
| HIV20SF1347 | OP191566 | 2020/10/16 |
| HIV20SF1394 | OP191567 | 2020/11/10 |
| HIV20SF1401 | OP191568 | 2020/11/14 |
| HIV20SF1417 | OP191569 | 2020/11/23 |
| HIV20SF1450 | OP191570 | 2020/12/7 |
| HIV20SF1489 | OP191571 | 2020/12/24 |
| HIV20SF1498 | OP191572 | 2020/12/28 |
| HIV21SF92 | OP191573 | 2021/2/27 |
| HIV21SF97 | OP191574 | 2021/3/1 |
| HIV21SF112 | OP191575 | 2021/3/18 |
| HIV21SF119 | OP191576 | 2021/4/12 |
| HIV22SF8 | PV568708 | 2022/3/2 |
| HIV22SF26 | PV568709 | 2022/3/11 |
| HIV22SF58 | PV568710 | 2022/3/23 |
| HIV22SF62 | PV568711 | 2022/3/28 |
| HIV22SF90 | PV568712 | 2022/4/6 |
| HIV22SF96 | PV568713 | 2022/4/12 |
| HIV22SF132 | PV568714 | 2022/4/27 |
| HIV22SF177 | PV568715 | 2022/5/30 |
| HIV22SF185 | PV568716 | 2022/6/2 |
| HIV22SF205 | PV568717 | 2022/6/14 |

## Slide 3
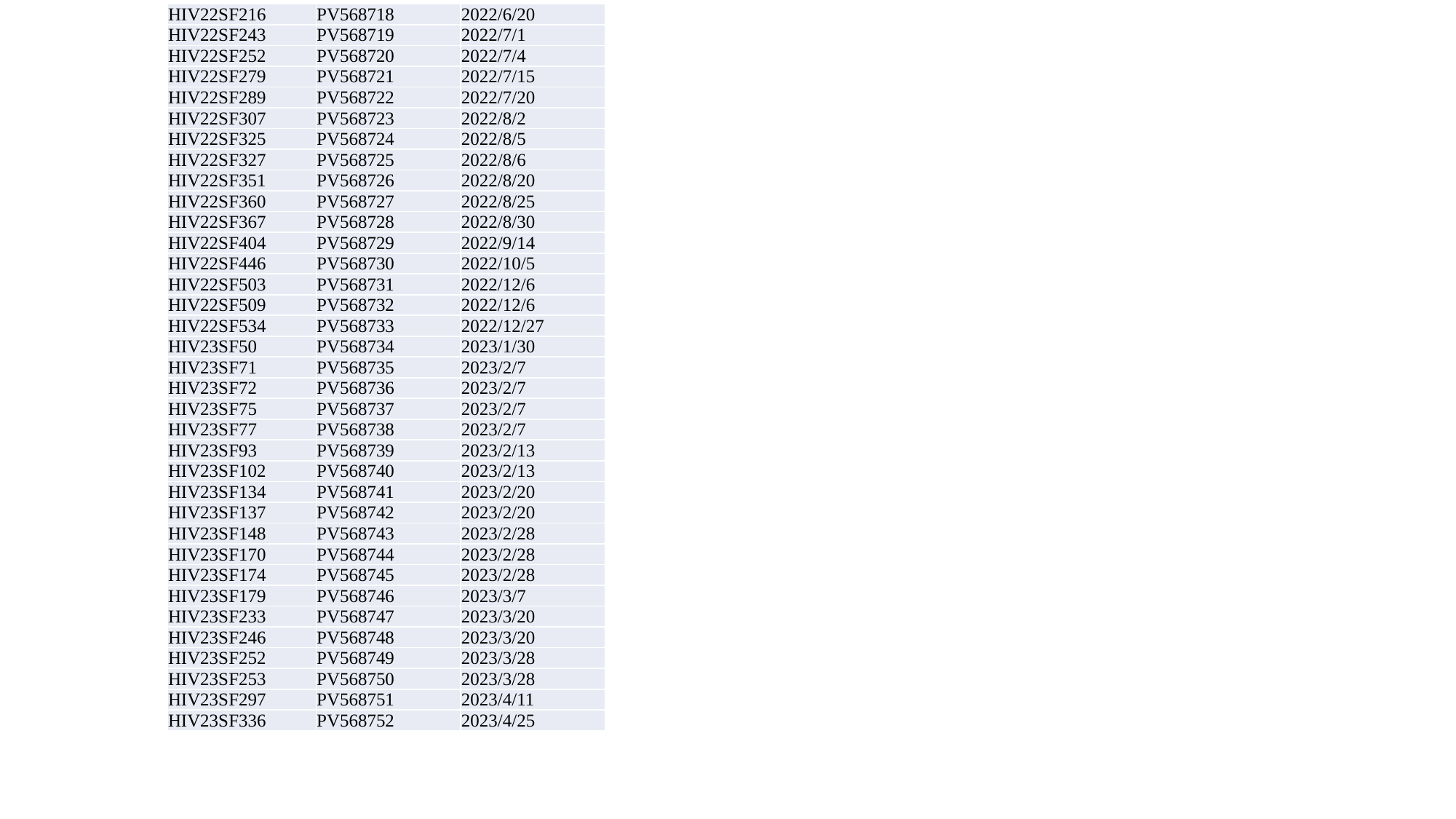

| HIV22SF216 | PV568718 | 2022/6/20 |
| --- | --- | --- |
| HIV22SF243 | PV568719 | 2022/7/1 |
| HIV22SF252 | PV568720 | 2022/7/4 |
| HIV22SF279 | PV568721 | 2022/7/15 |
| HIV22SF289 | PV568722 | 2022/7/20 |
| HIV22SF307 | PV568723 | 2022/8/2 |
| HIV22SF325 | PV568724 | 2022/8/5 |
| HIV22SF327 | PV568725 | 2022/8/6 |
| HIV22SF351 | PV568726 | 2022/8/20 |
| HIV22SF360 | PV568727 | 2022/8/25 |
| HIV22SF367 | PV568728 | 2022/8/30 |
| HIV22SF404 | PV568729 | 2022/9/14 |
| HIV22SF446 | PV568730 | 2022/10/5 |
| HIV22SF503 | PV568731 | 2022/12/6 |
| HIV22SF509 | PV568732 | 2022/12/6 |
| HIV22SF534 | PV568733 | 2022/12/27 |
| HIV23SF50 | PV568734 | 2023/1/30 |
| HIV23SF71 | PV568735 | 2023/2/7 |
| HIV23SF72 | PV568736 | 2023/2/7 |
| HIV23SF75 | PV568737 | 2023/2/7 |
| HIV23SF77 | PV568738 | 2023/2/7 |
| HIV23SF93 | PV568739 | 2023/2/13 |
| HIV23SF102 | PV568740 | 2023/2/13 |
| HIV23SF134 | PV568741 | 2023/2/20 |
| HIV23SF137 | PV568742 | 2023/2/20 |
| HIV23SF148 | PV568743 | 2023/2/28 |
| HIV23SF170 | PV568744 | 2023/2/28 |
| HIV23SF174 | PV568745 | 2023/2/28 |
| HIV23SF179 | PV568746 | 2023/3/7 |
| HIV23SF233 | PV568747 | 2023/3/20 |
| HIV23SF246 | PV568748 | 2023/3/20 |
| HIV23SF252 | PV568749 | 2023/3/28 |
| HIV23SF253 | PV568750 | 2023/3/28 |
| HIV23SF297 | PV568751 | 2023/4/11 |
| HIV23SF336 | PV568752 | 2023/4/25 |

## Slide 4
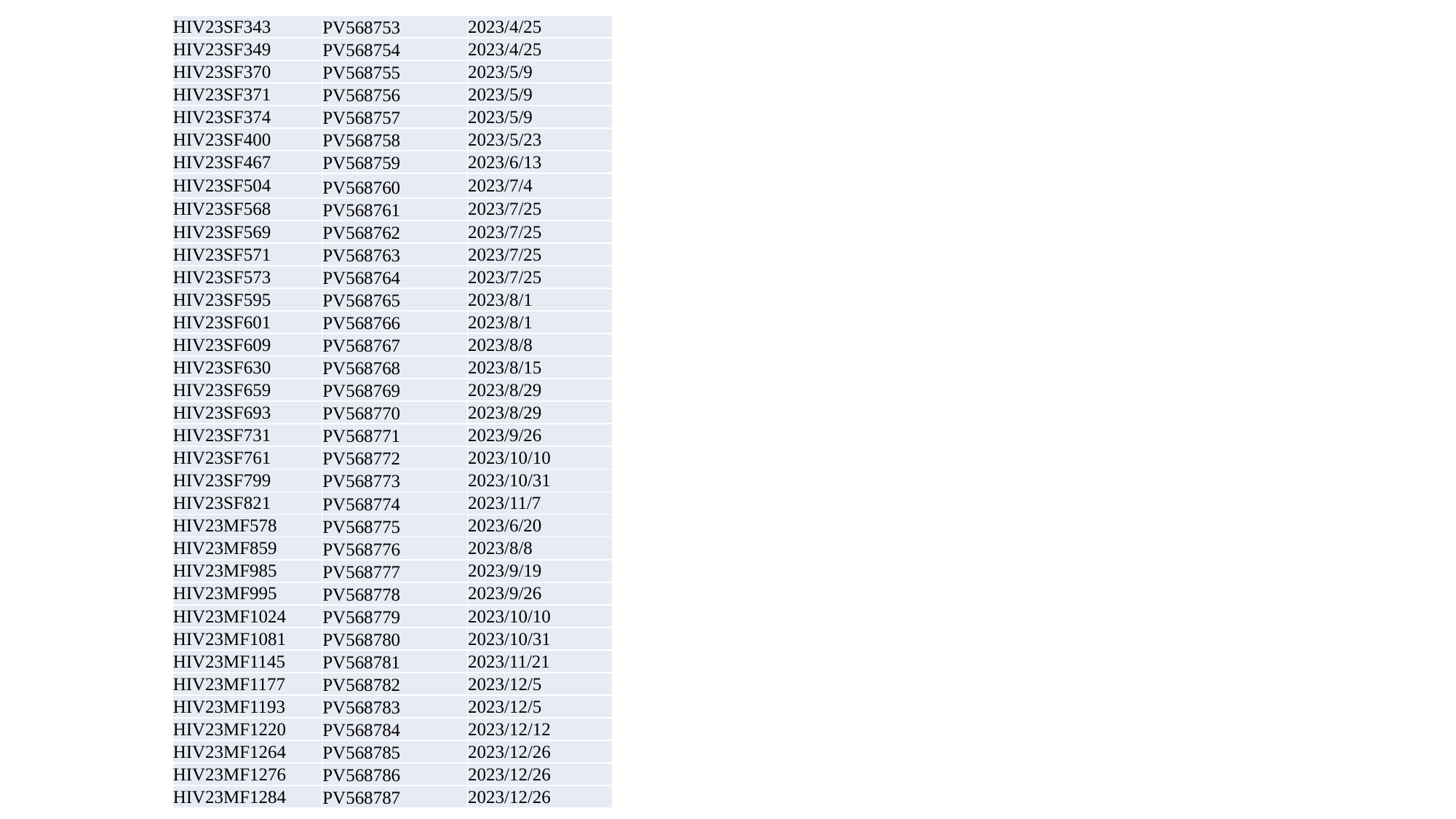

| HIV23SF343 | PV568753 | 2023/4/25 |
| --- | --- | --- |
| HIV23SF349 | PV568754 | 2023/4/25 |
| HIV23SF370 | PV568755 | 2023/5/9 |
| HIV23SF371 | PV568756 | 2023/5/9 |
| HIV23SF374 | PV568757 | 2023/5/9 |
| HIV23SF400 | PV568758 | 2023/5/23 |
| HIV23SF467 | PV568759 | 2023/6/13 |
| HIV23SF504 | PV568760 | 2023/7/4 |
| HIV23SF568 | PV568761 | 2023/7/25 |
| HIV23SF569 | PV568762 | 2023/7/25 |
| HIV23SF571 | PV568763 | 2023/7/25 |
| HIV23SF573 | PV568764 | 2023/7/25 |
| HIV23SF595 | PV568765 | 2023/8/1 |
| HIV23SF601 | PV568766 | 2023/8/1 |
| HIV23SF609 | PV568767 | 2023/8/8 |
| HIV23SF630 | PV568768 | 2023/8/15 |
| HIV23SF659 | PV568769 | 2023/8/29 |
| HIV23SF693 | PV568770 | 2023/8/29 |
| HIV23SF731 | PV568771 | 2023/9/26 |
| HIV23SF761 | PV568772 | 2023/10/10 |
| HIV23SF799 | PV568773 | 2023/10/31 |
| HIV23SF821 | PV568774 | 2023/11/7 |
| HIV23MF578 | PV568775 | 2023/6/20 |
| HIV23MF859 | PV568776 | 2023/8/8 |
| HIV23MF985 | PV568777 | 2023/9/19 |
| HIV23MF995 | PV568778 | 2023/9/26 |
| HIV23MF1024 | PV568779 | 2023/10/10 |
| HIV23MF1081 | PV568780 | 2023/10/31 |
| HIV23MF1145 | PV568781 | 2023/11/21 |
| HIV23MF1177 | PV568782 | 2023/12/5 |
| HIV23MF1193 | PV568783 | 2023/12/5 |
| HIV23MF1220 | PV568784 | 2023/12/12 |
| HIV23MF1264 | PV568785 | 2023/12/26 |
| HIV23MF1276 | PV568786 | 2023/12/26 |
| HIV23MF1284 | PV568787 | 2023/12/26 |

## Slide 5
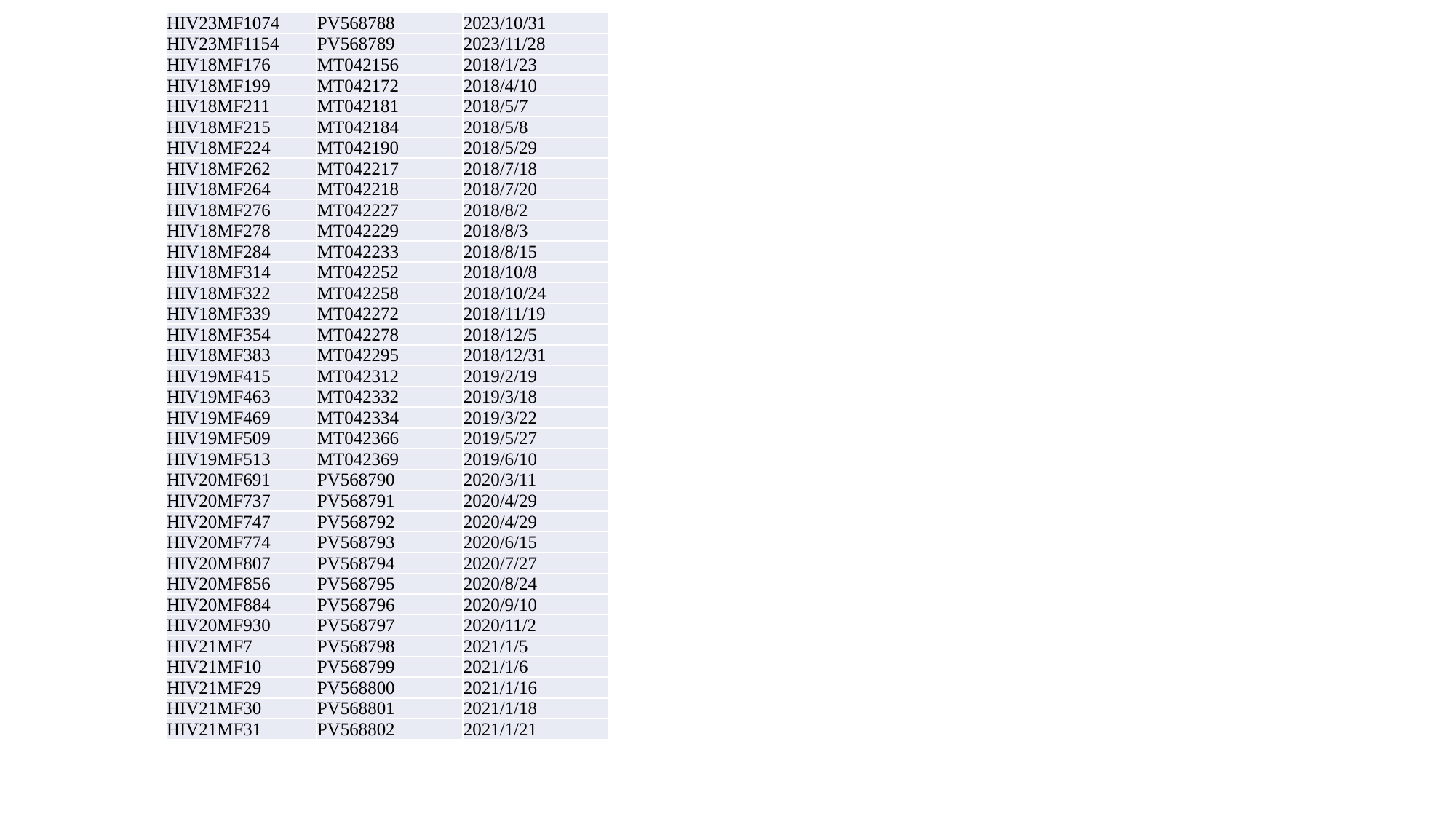

| HIV23MF1074 | PV568788 | 2023/10/31 |
| --- | --- | --- |
| HIV23MF1154 | PV568789 | 2023/11/28 |
| HIV18MF176 | MT042156 | 2018/1/23 |
| HIV18MF199 | MT042172 | 2018/4/10 |
| HIV18MF211 | MT042181 | 2018/5/7 |
| HIV18MF215 | MT042184 | 2018/5/8 |
| HIV18MF224 | MT042190 | 2018/5/29 |
| HIV18MF262 | MT042217 | 2018/7/18 |
| HIV18MF264 | MT042218 | 2018/7/20 |
| HIV18MF276 | MT042227 | 2018/8/2 |
| HIV18MF278 | MT042229 | 2018/8/3 |
| HIV18MF284 | MT042233 | 2018/8/15 |
| HIV18MF314 | MT042252 | 2018/10/8 |
| HIV18MF322 | MT042258 | 2018/10/24 |
| HIV18MF339 | MT042272 | 2018/11/19 |
| HIV18MF354 | MT042278 | 2018/12/5 |
| HIV18MF383 | MT042295 | 2018/12/31 |
| HIV19MF415 | MT042312 | 2019/2/19 |
| HIV19MF463 | MT042332 | 2019/3/18 |
| HIV19MF469 | MT042334 | 2019/3/22 |
| HIV19MF509 | MT042366 | 2019/5/27 |
| HIV19MF513 | MT042369 | 2019/6/10 |
| HIV20MF691 | PV568790 | 2020/3/11 |
| HIV20MF737 | PV568791 | 2020/4/29 |
| HIV20MF747 | PV568792 | 2020/4/29 |
| HIV20MF774 | PV568793 | 2020/6/15 |
| HIV20MF807 | PV568794 | 2020/7/27 |
| HIV20MF856 | PV568795 | 2020/8/24 |
| HIV20MF884 | PV568796 | 2020/9/10 |
| HIV20MF930 | PV568797 | 2020/11/2 |
| HIV21MF7 | PV568798 | 2021/1/5 |
| HIV21MF10 | PV568799 | 2021/1/6 |
| HIV21MF29 | PV568800 | 2021/1/16 |
| HIV21MF30 | PV568801 | 2021/1/18 |
| HIV21MF31 | PV568802 | 2021/1/21 |

## Slide 6
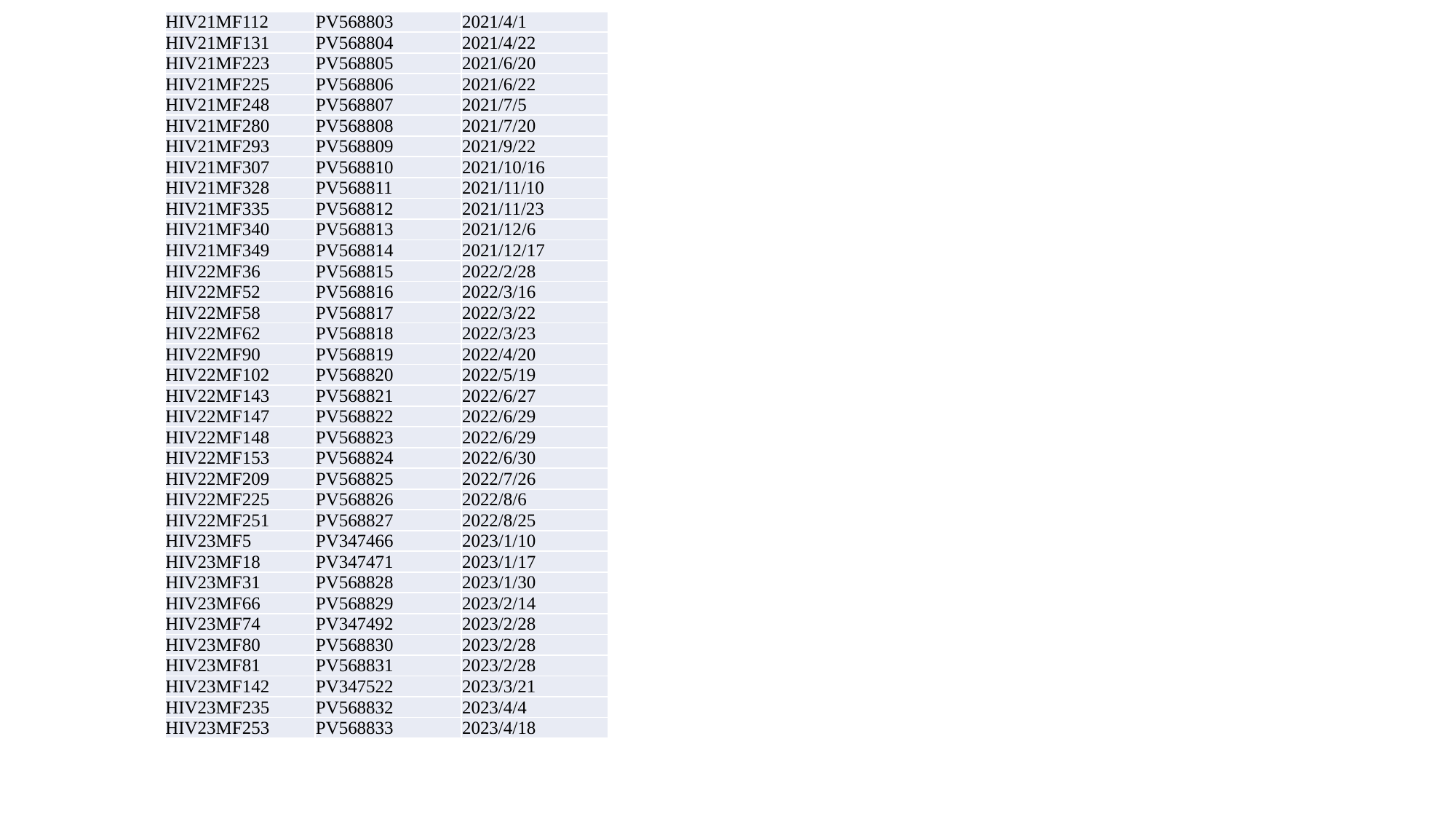

| HIV21MF112 | PV568803 | 2021/4/1 |
| --- | --- | --- |
| HIV21MF131 | PV568804 | 2021/4/22 |
| HIV21MF223 | PV568805 | 2021/6/20 |
| HIV21MF225 | PV568806 | 2021/6/22 |
| HIV21MF248 | PV568807 | 2021/7/5 |
| HIV21MF280 | PV568808 | 2021/7/20 |
| HIV21MF293 | PV568809 | 2021/9/22 |
| HIV21MF307 | PV568810 | 2021/10/16 |
| HIV21MF328 | PV568811 | 2021/11/10 |
| HIV21MF335 | PV568812 | 2021/11/23 |
| HIV21MF340 | PV568813 | 2021/12/6 |
| HIV21MF349 | PV568814 | 2021/12/17 |
| HIV22MF36 | PV568815 | 2022/2/28 |
| HIV22MF52 | PV568816 | 2022/3/16 |
| HIV22MF58 | PV568817 | 2022/3/22 |
| HIV22MF62 | PV568818 | 2022/3/23 |
| HIV22MF90 | PV568819 | 2022/4/20 |
| HIV22MF102 | PV568820 | 2022/5/19 |
| HIV22MF143 | PV568821 | 2022/6/27 |
| HIV22MF147 | PV568822 | 2022/6/29 |
| HIV22MF148 | PV568823 | 2022/6/29 |
| HIV22MF153 | PV568824 | 2022/6/30 |
| HIV22MF209 | PV568825 | 2022/7/26 |
| HIV22MF225 | PV568826 | 2022/8/6 |
| HIV22MF251 | PV568827 | 2022/8/25 |
| HIV23MF5 | PV347466 | 2023/1/10 |
| HIV23MF18 | PV347471 | 2023/1/17 |
| HIV23MF31 | PV568828 | 2023/1/30 |
| HIV23MF66 | PV568829 | 2023/2/14 |
| HIV23MF74 | PV347492 | 2023/2/28 |
| HIV23MF80 | PV568830 | 2023/2/28 |
| HIV23MF81 | PV568831 | 2023/2/28 |
| HIV23MF142 | PV347522 | 2023/3/21 |
| HIV23MF235 | PV568832 | 2023/4/4 |
| HIV23MF253 | PV568833 | 2023/4/18 |

## Slide 7
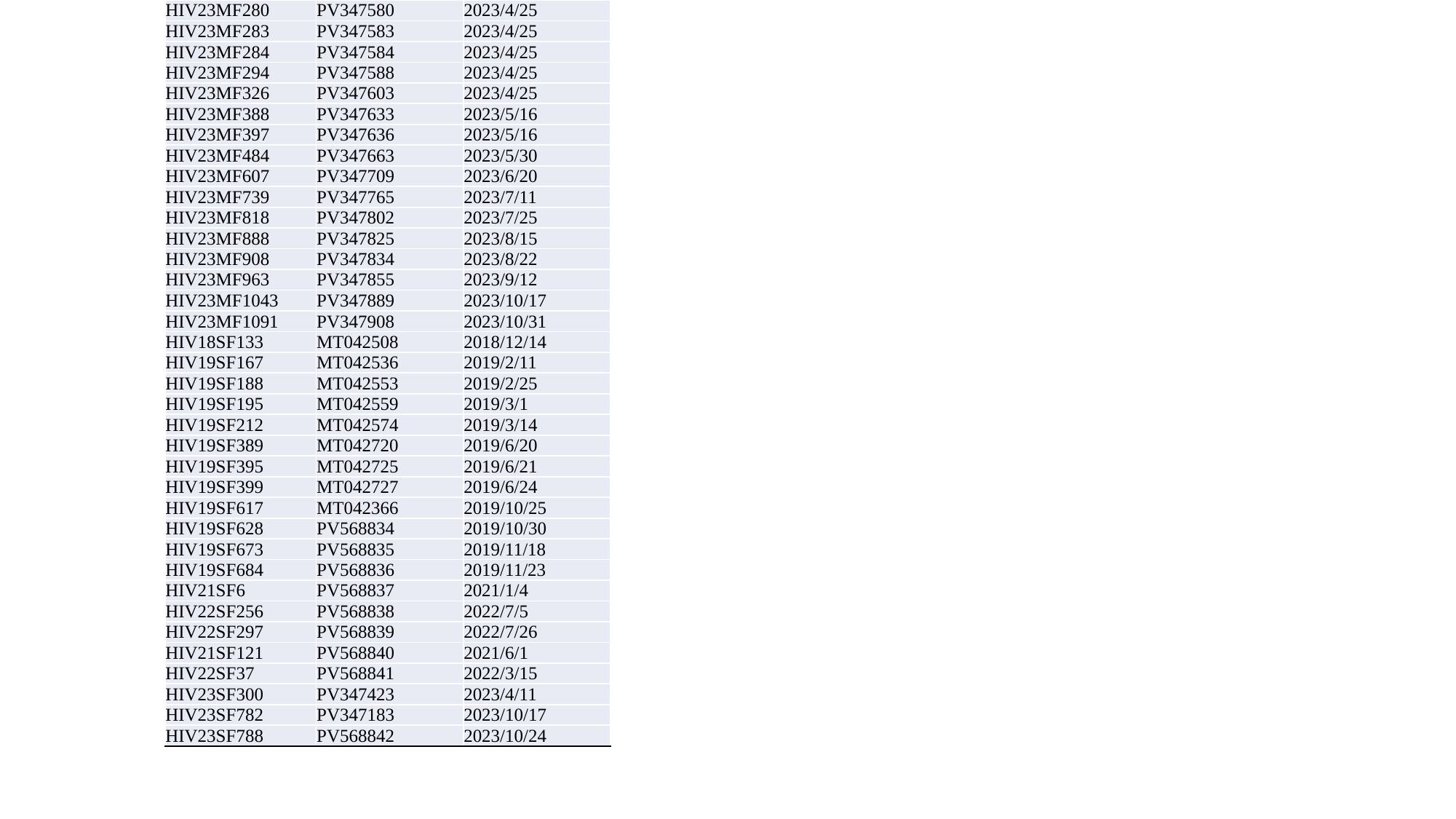

| HIV23MF280 | PV347580 | 2023/4/25 |
| --- | --- | --- |
| HIV23MF283 | PV347583 | 2023/4/25 |
| HIV23MF284 | PV347584 | 2023/4/25 |
| HIV23MF294 | PV347588 | 2023/4/25 |
| HIV23MF326 | PV347603 | 2023/4/25 |
| HIV23MF388 | PV347633 | 2023/5/16 |
| HIV23MF397 | PV347636 | 2023/5/16 |
| HIV23MF484 | PV347663 | 2023/5/30 |
| HIV23MF607 | PV347709 | 2023/6/20 |
| HIV23MF739 | PV347765 | 2023/7/11 |
| HIV23MF818 | PV347802 | 2023/7/25 |
| HIV23MF888 | PV347825 | 2023/8/15 |
| HIV23MF908 | PV347834 | 2023/8/22 |
| HIV23MF963 | PV347855 | 2023/9/12 |
| HIV23MF1043 | PV347889 | 2023/10/17 |
| HIV23MF1091 | PV347908 | 2023/10/31 |
| HIV18SF133 | MT042508 | 2018/12/14 |
| HIV19SF167 | MT042536 | 2019/2/11 |
| HIV19SF188 | MT042553 | 2019/2/25 |
| HIV19SF195 | MT042559 | 2019/3/1 |
| HIV19SF212 | MT042574 | 2019/3/14 |
| HIV19SF389 | MT042720 | 2019/6/20 |
| HIV19SF395 | MT042725 | 2019/6/21 |
| HIV19SF399 | MT042727 | 2019/6/24 |
| HIV19SF617 | MT042366 | 2019/10/25 |
| HIV19SF628 | PV568834 | 2019/10/30 |
| HIV19SF673 | PV568835 | 2019/11/18 |
| HIV19SF684 | PV568836 | 2019/11/23 |
| HIV21SF6 | PV568837 | 2021/1/4 |
| HIV22SF256 | PV568838 | 2022/7/5 |
| HIV22SF297 | PV568839 | 2022/7/26 |
| HIV21SF121 | PV568840 | 2021/6/1 |
| HIV22SF37 | PV568841 | 2022/3/15 |
| HIV23SF300 | PV347423 | 2023/4/11 |
| HIV23SF782 | PV347183 | 2023/10/17 |
| HIV23SF788 | PV568842 | 2023/10/24 |
